# Renal Effects of DPP-4 Inhibitors: A Focus on Microalbuminuria

**DOI:** 10.1155/2013/895102

**Published:** 2013-09-05

**Authors:** Martin Haluzík, Jan Frolík, Ivan Rychlík

**Affiliations:** ^1^Third Department of Medicine, First Faculty of Medicine, Charles University and General University Hospital, U nemocnice 1, 120 00 Prague, Czech Republic; ^2^Eli Lilly and Company, Pobrezni 12, 180 00 Prague, Czech Republic; ^3^Third Faculty of Medicine, Charles University, Srobarova 50, 100 34 Prague, Czech Republic

## Abstract

Incretin-based therapies represent one of the most promising options in type 2 diabetes treatment owing to their good effectiveness with low risk of hypoglycemia and no weight gain. Other numerous potential beneficial effects of incretin-based therapies have been suggested based mostly on experimental and small clinical studies including its beta-cell- and vasculo-protective actions. One of the recently emerged interesting features of dipeptidyl peptidase-4 (DPP-4) inhibitors is its possible protective effect on the diabetic kidney disease. Here, we review the renal effects of DPP-4 inhibitors with special focus on its influence on the onset and progression of microalbuminuria, as presence of microalbuminuria represents an important early sign of kidney damage and is also associated with increased risk of hypoglycemia and cardiovascular complications. Mechanisms underlying possible nephroprotective properties of DPP-4 inhibitors include reduction of oxidative stress and inflammation and improvement of endothelial dysfunction. Effects of DPP-4 inhibitors may be both glucagon-like peptide-1 (GLP-1) dependent and independent. Ongoing prospective studies focused on the nephroprotective effects of DPP-4 inhibitors will further clarify its possible role in the prevention/attenuation of diabetic kidney disease beyond its glucose lowering properties.

## 1. Introduction

Increasing prevalence of diabetes worldwide, leading to a steep rise of patients with chronic complications, represents one of the major health problems of the current medicine [1]. Since both micro- and macrovascular complications contribute in the increasing morbidity and mortality of patients with type 2 diabetes, novel antidiabetic therapies are intensively studied with respect to their possible beneficial effects on the long-term complications beyond their glucose-lowering properties [2]. Incretin-based therapies represent one of the most promising options in type 2 diabetes treatment owing to their good effectiveness with low risk of hypoglycemia and no weight gain [3]. These therapeutics either increase concentrations of endogenous glucagon-like peptide-1 (GLP-1) by the inhibition of its degradation (dipeptidyl peptidase-4 inhibitors) or directly stimulate GLP-1 receptor (GLP-1 receptor agonists) [4]. Stimulation of GLP-1 receptor in turn increases insulin secretion and suppresses excessive glucagon release leading to improved glucose control. Other numerous potential beneficial effects of incretin-based therapies have been suggested based mostly on experimental and small clinical studies including its beta-cell- and vasculoprotective actions and also numerous others pleiotropic positive effects such as neuroprotection and others [5]. One of the interesting possibilities that have emerged from experimental studies is the protective effect of DPP-4 inhibitors on the diabetic kidney disease [6]. Here, we review the renal effects of DPP-4 inhibitors with special focus on its influence on the onset and progression of microalbuminuria. We will discuss potential mechanism of these effects, the differences between various DPP-4 inhibitors, and future perspectives of its use in patients with diabetic kidney disease. We performed a primary Medline search using combinations of keywords: sitagliptin, vildagliptin, saxagliptin, linagliptin, exenatide, liraglutide, and GLP-1, DPP-4 with albuminuria, and we consequently used all relevant articles published in English language in this review. Due to a limited number of results, we performed secondary searches using combinations of additional keywords like diabetic kidney disease and nephropathy.

## 2. Diabetic Kidney Disease: Basic Pathophysiology

Diabetic kidney disease (DKD) represents one of the most frequent microvascular complications of diabetes with an overall prevalence of approximately 40% in type 2 diabetes population [7]. DKD is defined by the presence of albuminuria and decreased glomerular filtration rate (GFR) into 5 chronic kidney disease (CKD) stages. CKD stage 1 is characterized by normal GFR and urine findings (mostly albuminuria) or structural abnormalities of the kidney. Stages 2–5 are defined by specific values of GFR [7]. Patients with diabetic kidney disease, even in stage 1, have a markedly increased risk of cardiovascular complications and hypoglycemia compared to patients without DKD [8, 9]. Numerous studies have shown that the risk of diabetic kidney disease is tightly linked to poor glucose control in both type 1 and type 2 diabetes [10, 11]. The adverse effects of hyperglycemia are generally mediated through diverse metabolic pathways including increased reactive oxygen species formation, excessive production of advanced glycation end products (AGEs), and the activation of polyol, protein kinase C (PKC), and hexosamine pathways, respectively [12]. Activation of these pathways leads to a complex dysregulation of various effector molecules resulting in cellular damage and dysfunction [12]. Experimental studies have shown that some of these pathophysiological mechanisms are potentially modifiable by DPP-4 inhibition [6]. Activation of PKC in the kidney by hyperglycemia reduces GLP-1 signaling while enhancing angiotensin II and nuclear factor-*κ*B (NF-*κ*B) signaling pathways with subsequent development of glomerular endothelial dysfunction [12]. This process that typically occurs in the early stages of diabetic kidney disease is characterized by decreased local production of nitric oxide and enhanced production of reactive oxygen species, proinflammatory and cell adhesive molecules including tumor necrosis factor-*α* (TNF-*α*), plasminogen activator inhibitor-1 (PAI-1), intercellular adhesion molecule-1 (ICAM-1), vascular cell adhesion molecule-1 (VCAM-1), and others [13]. Increased expression of ICAM-1 on glomerular endothelial cells promotes local macrophage infiltration into glomeruli with subsequent development of local inflammation and increased production of the renal profibrotic cytokine transforming growth factor *β* (TGF-*β*) [14]. Chronic overproduction of TGF-*β* markedly contributes to local degenerative changes and progressive fibrosis in diabetic kidney [14]. Additional important players contributing to kidney damage especially in patients with type 2 diabetes include arterial hypertension and dyslipidemia that commonly cluster with glucose metabolism disturbances in these patients [15].

## 3. Mechanism of Albuminuria

The presence of microalbuminuria represents an important early sign of kidney damage in patients with diabetes [16]. Associations between microalbuminuria, increased risk of cardiovascular complications, and progressive renal impairment are well described but the underlying pathophysiological mechanisms are only partially understood [16]. In general, the presence of microalbuminuria implies either dysfunction of the glomerular filtration barrier and/or dysfunction in tubular reabsorption [17]. At the time of overt microalbuminuria, established glomerular structural changes are present in diabetic kidney [18]. Studies have shown that local changes in glomerular morphology and the extent of matrix accumulation in glomeruli and interstitium correlate with extent of albuminuria [19]. Glomerular filtration barrier is characterized by three-layer structure: endothelium with glycocalyx, glomerular basement membrane, and podocytes [12]. Endothelial glycocalyx forms a barrier to protein permeability in both systemic and glomerular capillaries. The total systemic glycocalyx volume is reduced by acute hyperglycemia in humans [20]. Podocyte loss is one of the first changes contributing to increased glomerular permeability for albumin. Nevertheless, albuminuria may also occur in the complete absence of structural changes of podocytes [21, 22]. Tubular dysfunction is another important player promoting albuminuria by lysosomal dysfunction which is promoted by renin-angiotensin system activation and TGF-*β* overproduction [23]. A number of experimental studies demonstrated that TGF-*β* neutralization improved renal function and reversed morphological changes associated with disease progression in diabetic nephropathy models [24]. TGF-*β* is typically elevated in serum, urine, and glomerular tissue since early stages of both type 1 and 2 diabetes. Its levels correlate with the degree of mesangial expansion, interstitial fibrosis, and renal dysfunction, but not with the extent of microalbuminuria [25]. On the contrary, urinary levels of TNF-*α* and vascular endothelial growth factor (VEGF) tightly correlate with microalbuminuria [26, 27]. Both of these cytokines are upregulated in patients with type 2 diabetes [26, 27]. TNF-*α* directly increases endothelial permeability and disrupts the endothelial glycocalyx [27]. Albuminuria also positively correlates with markers of endothelial dysfunction and chronic low-grade inflammation including C-reactive protein [28, 29].

## 4. GLP-1 Dependent and Independent Actions of DPP-4 Inhibitors in the Kidney

The main action of DPP-4 inhibitors is to increase the levels of endogenous incretin hormones, especially GLP-1. DPP-4 inhibitors are known to exert also GLP-1 independent effects as DPP-4 cleaves a wide range of other substrates such as neuropeptides, hormones, cytokines, and chemokines [6, 30]. DPP-4 is also bound on the surface of many cell types including kidney proximal tubular cells and endothelial cells [31]. Microvesicle-bound DPP-4 secreted from tubular epithelial cells is found in urine and may be an early marker of renal damage before the onset of albuminuria [31]. Sun et al. also described higher urinary microvesicle DPP-4 levels in patients with diabetes compared to nondiabetic controls that positively correlated with extent of albuminuria in patients [31]. Upregulation of DPP-4 expression in renal glomeruli occurs during inflammation and usually accompanies the development of diabetes-induced glomerulosclerosis [6].

Renal effects of DPP-4 inhibitors appear to be, at least in part, mediated by increased GLP-1 levels [32]. In addition to pancreas, GLP-1 receptor (GLP-1R) is expressed in other numerous tissues including glomerular endothelial cells, mesangial cells, podocytes and also proximal tubular cells. Its expression was decreased in diabetic compared with nondiabetic mice [32]. Studies have shown that GLP-1 has anti-inflammatory properties and decreases AGEs production by activation of protein kinase A (PKA). The stimulation of nitric oxide (NO) production and inhibition of production of angiotensin II, PAI-1, ICAM-1, and VCAM-1 seems to be both GLP-1 dependent and independent [33–37].

Several candidates for GLP-1 independent effects of DPP-4 inhibitors in the kidney have been identified among known substrates of DPP-4 cleavage including high mobility group protein-B1 (HMGB1), Meprin *β*, neuropeptide Y (NPY), and peptide YY (PYY). Nevertheless, their exact role and importance in renoprotective effects of DPP-4 inhibitors has not yet been tested [38]. HMGB1 is a known ligand of advances glycation end products receptor (RAGE), as well as Toll-like receptor 2 (TLR2) and TLR4, which are involved in the inflammatory process of diabetic nephropathy leading to NF-*κ*B activation [38]. Meprin *β* has been associated with several types of renal pathology [39]. Both NPY and PYY are important mediators of various kidney functions including natriuresis [40–44]. Experimental studies have also shown that DPP-4 participates in the extracellular catabolism of proteins in the kidney such as catabolism/degradation proline-containing peptides [45].

## 5. Preclinical Data of DPP-4 Inhibitors with Nephroprotective Outcomes

Preclinical data suggesting nephroprotective effects of DPP-4 inhibitors are available for sitagliptin [46], vildagliptin [47], and linagliptin [48]. The study with sitagliptin administration assessed its effects on metabolic profile and renal lesions in a rat model of type 2 diabetic nephropathy [46]. Diabetic and controls rats were treated with sitagliptin or vehicle for 6 weeks. Sitagliptin treatment of diabetic rats lowered glycemia and ameliorated glomerular, tubulointerstitial, and vascular lesions. It also reduced kidney lipid peroxidation as measured by decreased malondialdehyde content.

The study with vildagliptin was performed on rats with streptozotocin-induced diabetes. In this insulinopenic model, vildagliptin increased GLP-1 levels but did not affect blood glucose levels [47]. Light and electron microscopies of renal tissue revealed that vildagliptin treatment dose dependently inhibited interstitial expansion, glomerulosclerosis, and thickening of the glomerular basement membrane [47]. It also significantly decreased both albuminuria and proteinuria and reduced TGF-*β* overexpression. Expression of GLP-1R was demonstrated by immunohistochemical analysis in both glomeruli and tubules. The 24-week duration of hyperglycemia in untreated diabetic rats resulted in decrease of GLP-1R staining [47]. This decrease was prevented by treatment with vildagliptin. Furthermore, increased urinary excretion rates of 8-Oxo-2′-deoxyguanosine (major product of DNA oxidation and marker of oxidative stress) in diabetic rats were markedly attenuated by vildagliptin treatment. These results suggest prevention of oxidative DNA damage and renal cell apoptosis by activating the GLP-1R.

Streptozotocin-induced diabetes was used also in an experimental study with linagliptin [48]. Diabetes was induced in endothelial nitric oxide synthase (eNOS) knockout mice which were used as an experimental model of nephropathy [48]. The effect of linagliptin on the progression of nephropathy alone and in combination with the angiotensin receptor blocker telmisartan was tested. After 12 weeks of administration, linagliptin or telmisartan had no effect on glycemic control, while telmisartan reduced systolic blood pressure by 5.9 mmHg compared to no change in linagliptin group. Combined treatment with linagliptin and telmisartan significantly reduced albuminuria compared with untreated diabetic mice, while monotherapy with either telmisartan or linagliptin had no effect. Reduced glomerulosclerosis and normalization of tissue immune reactivity of malondialdehyde, a biomarker of oxidative stress, were seen in linagliptin and combined linagliptin/telmisartan group while angiotensin II receptor blockade alone failed to reduce the markers of oxidative stress.

Another data suggesting possible nephroprotective effects of DPP-4 inhibitors come from the study on the experimental model of renal ischemia/reperfusion injury [49]. In this study, vildagliptin was administered intravenously 15 minutes before surgery, and animals were sacrificed after 2, 12, and 48 hours of reperfusion. DPP-4 inhibition dose dependently decreased serum creatinine, tubular necrosis, serum malondialdehyde levels, and mRNA expression of proinflammatory chemokine CXCL10. These data suggest that the nephroprotection by DPP-4 inhibition was mediated by antiapoptotic, anti-inflammatory, and antioxidative changes.

In addition to experimental data for DPP-4 inhibitors, animal studies suggest also possible nephroprotective effects of GLP-1R agonists. Both exendin-4 [35] and liraglutide [50] ameliorated albuminuria decreased oxidative stress and inflammatory cytokines in a rat model of diabetic nephropathy. In the exendin-4 study, glomerular macrophage infiltration was prevented by suppression of ICAM-1 production on glomerular endothelial cells and by inhibition of proinflammatory cytokine release from macrophages.

## 6. Clinical Studies of DPP-4 Inhibitors with Albuminuria Outcomes

Many diabetic patients develop diabetic kidney disease despite intensive efforts to achieve optimal control of blood pressure and glycemia. In addition to being a marker of renal damage, albuminuria has emerged as a predictive marker of increased risk of cardiovascular disease [51]. According to current guidelines, the primary intervention in patients with detected albuminuria is the blockade of renin-angiotensin-aldosterone system (RAAS) with an angiotensin-converting enzyme inhibitor (ACEi) or angiotensin II receptor blocker (ARB) [52]. Proven additive effect of any antihyperglycemic agent on albuminuria lowering would be therefore of a high interest.

First study indicating possible beneficial effect of a DPP-4 inhibitor on the kidney in Man was a small observational study with sitagliptin [53]. 36 patients with HbA1c > 6.5%, despite lifestyle measures and antidiabetic treatment for at least 6 months, were enrolled and treated by sitagliptin (50 mg/day) for 6 months. Sitagliptin significantly lowered HbA1c and systolic and diastolic blood pressure. Significant reductions of C-reactive protein and soluble VCAM-1 were also observed. After 6 months of sitagliptin treatment, albuminuria measured by urinary albumin creatinine ratio (UACR) significantly decreased both in the patients with relatively modest microalbuminuria and the patients with more pronounced microalbuminuria at baseline. Albuminuria was also one of secondary endpoints in the study of Harashima et al. performed with sitagliptin [54]. 82 subjects were enrolled to the 52-week, prospective, single-arm study where sitagliptin was added-on to sulphonylureas (glimepiride or gliclazide) with or without metformin. The primary endpoint was a change in HbA1c. The secondary endpoints were changes in BMI, insulin secretory capacity, blood pressure, UACR, unresponsive rate, and hypoglycemia. After 52 weeks sitagliptin treatment reduced HbA1c by 0.8% and UACR from 76.2 ± 95.6  to 33.0 ± 48.1 mg/g along with slight decreases in BMI blood pressure.

In 2012, a comprehensive analysis of randomized, double-blind, placebo-controlled trials (duration 24–52 weeks) with linagliptin was published [55]. The analysis included studies with both linagliptin monotherapy or add-on therapy to various glucose-lowering agents. Inclusion criteria for the analysis were diabetic patients with persistent albuminuria defined as 30 ≤ UACR ≤ 3000 mg/g and stable treatment with ACE inhibitor or angiotensin II receptor blocker at baseline. The analysis included 168 patients treated with linagliptin and 59 patients on placebo, respectively. The endpoint of the analysis was the percentage change in geometric mean of UACR after 24 weeks of treatment compared to baseline values. Placebo-corrected reduction of HbA1c reached −0.71% blood pressure, and renal function remained unchanged. In linagliptin-treated group, UACR significantly decreased by 33% with a between-group difference versus placebo of −29%. Interestingly, the degree of microalbuminuria reduction did not correlate with the magnitude of change in HbA1c suggesting that the effects may have been independent of improvement in glycemic control.

In a recently published meta-analysis of 13 linagliptin trials including 5466 patients focused on composite renal outcome, the hazard ratio of 0.84 in favor of linagliptin compared to placebo or comparator was found [56]. The risk ratios for individual renal endpoints were 0.85 for microalbuminuria, 0.88 for macroalbuminuria, 0.44 for new onset of DKD, 0.77 for worsening of DKD, 0.93 for acute renal failure, and 0.77 for death, respectively.

Although none of the studies was primarily designed to test the effect of linagliptin on microalbuminuria and renal function, they collectively suggest its possible nephroprotective effects. Furthermore, two small studies with sitagliptin and the experimental studies described previously open the possibility that nephroprotection might be a class effect of DPP-4 inhibition. On the other hand, DPP-4 inhibitors have different pharmacokinetics, and their nonglucose lowering effects may vary in man due to different concentrations in various organs and due to distinct substrate selectivity of binding to DPP-4 enzyme [57].

## 7. Clinical Studies of Other Glucose Lowering Agents with Albuminuria Outcomes

In general, all long-term studies with antidiabetic agents suggest that good glucose control can prevent or delay the development of microvascular complications in both type 1 and type 2 diabetes [10, 11]. In short-term studies, the specific influence of different antidiabetic medications on microalbuminuria might differ substantially. In a small 16 weeks study comparing GLP-1R agonist exenatide with glimepiride, no differences in improvement of glucose control were found but a 24-hour urinary albumin was reduced by 40% in exenatide group compared to 5% reduction in glimepiride group. Furthermore, urinary TGF-*β* and type IV collagen in the exenatide group were also significantly reduced compared to no change in glimepiride-treated group [58]. Another antidiabetic medication affecting microalbuminuria is thiazolidinediones that share several mechanisms of action with incretin-based therapies including amelioration of increased activation of protein kinase C pathway in glomerular mesangial cells, improvement of impaired endothelial function, inhibition of mesangial and tubular cell proliferation by suppressing TGF-*β* expression, anti-inflammatory actions by attenuation of interleukin-1, 6, and TNF-alpha in renal mesangial cells, and reduction of oxidative stress in the kidney [59, 60]. In a 52-week, open-label, cardiac safety study comparing rosiglitazone to glyburide, only the rosiglitazone group showed a significant reduction of albuminuria from baseline [59]. For patients with microalbuminuria at baseline, reductions in UACR did not correlate with reductions in HbA1c or fasting plasma glucose but showed strong correlation with changes in mean 24 h systolic and diastolic blood pressures in rosiglitazone-treated patients. Another small randomized study comparing pioglitazone versus metformin in patients with baseline albuminuria treated with RAAS blockade showed similar effects [60]. After 52 weeks of treatment, the changes in UACR from baseline were −8.3% in pioglitazone group and +4.2% in metformin group (*P* = 0.01) with similar glycemic and blood pressure changes in both arms. These results suggest that metformin does not share the albuminuria-lowering potential of thiazolidinediones and incretin-based therapies [60].

Collectively, these data suggest a possibility of specific and glucose-lowering independent effects of incretin-based therapies and thiazolidinediones on the renal damage in patients with diabetes. Nevertheless, larger trials designed primary on testing renal outcomes are necessary to confirm this interesting possibility.

## 8. Conclusion and Perspectives

The results of published preclinical and clinical studies suggest that DPP-4 inhibitors may have a potential to lower albuminuria and to also possess other more complex nephroprotective properties. Possible mechanisms underlying these effects include reduction of oxidative stress and inflammation and improvement of endothelial dysfunction in the kidney. Effects of DPP-4 inhibitors may be both GLP-1 dependent and GLP-1 independent. At the moment, the data are too scarce and incomplete to make definite conclusions with respect to DPP-4 inhibitors induced nephroprotection. Ongoing studies, such as the MARLINA study comparing, prospectively, the effects of linagliptin to placebo on albuminuria may shed some light in this field [61]. Furthermore, numerous ongoing long-term cardiovascular trials with DPP-4 inhibitors can bring novel crucial information about relationships among glucose control and macrovascular and microvascular complications and further elucidate the role of albuminuria in these processes.

## Abbreviations

ACEi: Angiotensin-converting enzyme inhibitor
AGEs: Advanced glycation end-products
ARB: Angiotensin II receptor blocker
CKD: Chronic kidney disease
DKD: Diabetic kidney disease
DPP-4: Dipeptidyl peptidase-4
eNOS: Endothelial nitric oxide synthase
GFR: Glomerular filtration rate
GLP-1: Glucagon like peptide-1
HMGB1: High mobility group protein-B1
ICAM-1: Intercellular adhesion molecule-1
GLP-1R: GLP-1 receptor
NF-*κ*B: Nuclear factor-*κ*B
NO: Nitric oxide
NPY: Neuropeptide Y
PAI-1: Plasminogen activator inhibitor-1
PKA: Protein Kinase A
PKC: Protein Kinase C
PYY: Peptide YY
RAAS: Renin-angiotensin-aldosterone system
RAGE: Advances glycation endproducts receptor
TGF-*β*: Transforming growth factor *β*
TLR-2: Toll-like receptor 2
TLR-4: Toll-like receptor 4
TNF-*α*: Tumor necrosis factor-*α*
UACR: Urinary albumin creatinine ratio
VCAM-1: Vascular cell adhesion molecule-1
VEGF: Vascular endothelial growth factor.
